# Association of Salivary Microbial, Fungal Population and Salivary Acidity with Obesity in Children 

**DOI:** 10.30476/dentjods.2024.102462.2364

**Published:** 2025-09-01

**Authors:** Aida Mehdipour, Mojtaba Hossein Nattaj, Roohollah Fateh, Mohammad Aghaali, Romina Qomeisi, Ali Saleh, Mohammad Hassan Kalantar Neyestanaki

**Affiliations:** 1 Cellular and Molecular Research Center, Qom University of Medical sciences, Qom, Iran.; 2 Dept. of Pediatric Dentistry, Dental Faculty, Qom University of Medical Sciences, Qom, Iran.; 3 Dept. of Prosthodontics, Dental Faculty, Qom University of Medical Sciences, Qom, Iran.; 4 Dept. of Community Medicine, School of Medicine, Qom University of Medical Sciences, Qom, Iran.; 5 Student Research Committee, Qom university of Medical Sciences, Qom, Iran.; 6 Medical Doctor, School of Medicine, Arak University of Medical Sciences, Arak, Iran.

**Keywords:** Body Mass Index, Saliva, *Candida albicans*, *Streptococcus mutans*, *Lactobacillus*

## Abstract

**Background:**

Childhood obesity is an increasing global health concern associated with both systemic and oral complications. While studies suggest links between body mass index (BMI) and oral health markers, these relationships remain poorly defined.

**Purpose:**

This study evaluated the association between salivary microbial/fungal populations, salivary acidity, and obesity in children.

**Materials and Method:**

In this cross-sectional descriptive study (2021–2022), 90 children aged 8-12 from public schools in Qom, Iran, were categorized into three groups based on BMI: 30 children with normal weight, 30 overweight children, and 30 obese children. Demographic information, including parental education and occupation, was recorded. Unstimulated saliva samples were collected using the passive drooling method. A total of 0.5 ml of saliva was mixed with 5 ml of phosphate-buffered saline (PBS) and homogenized thoroughly by using a shaker. Microbiological analysis involved quantifying colonies of *Streptococcus mutans, Lactobacillus,* and *Candida albicans* and measuring salivary pH using a calibrated pH meter. Data were analyzed using appropriate statistical tests with significance set at *p Value*< 0.05.

**Results:**

No significant relationship was found between childhood obesity and parental education (father’s *p*= 0.051, mother’s *p*= 0.862) or occupation (father’s *p*= 0.224, mother’s *p*= 0.893). Salivary pH did not differ significantly between weight groups (*p*= 0.639). Overweight children had lower *Lactobacillus* levels (*p*= 0.857), and obese children had higher *Streptococcus mutans* levels (*p*= 0.777); though neither correlated significantly with BMI. *Candida albicans* colonies showed a significant negative correlation with childhood obesity (*p*= 0.046). Significant associations were also observed between *Streptococcus mutans* (*p*= 0.046) and Lactobacillus (*p*= 0.002) levels with *Candida albicans* levels.

**Conclusion:**

Although oral bacterial levels did not differ significantly across weight groups, fungal species, particularly *Candida albicans*, varied significantly. A negative association between obesity and *Candida albicans* counts suggests that obesity may impact the salivary microbial ecosystem, highlighting the need for further research into its systemic and oral health implications.

## Introduction

Obesity is defined as an abnormal accumulation of fat in one’s tissues [ 1 ]. Obesity is considered not only an aesthetic problem but also a chronic disease generated by the interrelationship between genetic, environmental, socio-economic, and behavioral components [ 2 ]. The prevalence of obesity has increased over the past 50 years globally and has turned into a major health problem [ 3 ]. According to a report released by the World Health Organization (WHO), more than 340 million children and adolescents aged 5-19 years were either overweight or obese [ 4 ]. Overall, the prevalence of obesity has increased worldwide in the past decade, attributed to many factors such as increased fat intake, consumption of processed foods and sugary beverages, reduced physical activity, and limited opportunities for exercise among children [ 5 ]. Akbari *et al*. [ 6 ] (2022) has reported the overall prevalence of obesity among Iranian children to be 11.4%. 

Body mass index (BMI) is an easy reliable method related to one’s body fat percentage. It can also be used to assess the risk of obesity-associated complications and mortality in adults [ 7 ]. 

Oral bacteria, through carbohydrate metabolism, lead to acid production, demineralization, and tooth decay. The main bacterial species involved in this process are *Streptococcus mutans* and *Lactobacillus* [ 8 ]. *Streptococcus mutans* is an acidogenic, aciduric bacterium and a primary pathogen in changing the oral pH [ 9 ]. *Lactobacillus*, constituting approximately 1% of the oral microbial flora, is highly aciduric and can survive at a pH=5.5 [ 10 ]. Some studies have reported an association between obesity and dental caries [ 11 ]. This might be due to the consumption of high-sugar snacks, resulting in both obesity and tooth decay [ 12 - 13 ]. A study conducted in 2021 showed a positive correlation between BMI percentile, the number of *Streptococcus mutans* and *Lactobacillus* in saliva, and the occurrence of dental caries among 9- to 12-year-old children [ 14 ]. The most common fungal infections of the oral cavity include candidiasis, aspergillosis, mucormycosis (zygo-mycosis), histoplasmosis, blastomycosis, cryptococcosis, paracoccidioidomycosis, and geotrichosis. Species of the* Candida* genus, such as *Candida albicans*, *Candida dubliniensis*, and *Candida tropicalis*, are fungi commonly found in mucosal niches and are frequently identified in biofilms on the teeth of toddlers with severe early childhood caries. *Candida albicans* isolates exhibit broad phenotypic variation but consistently display cariogenic traits, including high proteinase activity, acidogenicity, and acid tolerance. Notably, *Candida albicans* isolates show altered transcriptomes related to pH, adhesion, and cell wall composition compared to reference strains, further supporting their niche-associated traits [ 15 ]. Additionally, *in vitro* and animal studies have demonstrated that *Candida albicans* colonization increases the cariogenicity of oral biofilms by altering microbial ecology and influencing other oral bacteria [ 13 , 16 ]. Furthermore, there is a direct relationship between obesity and an increased population of bacteria in one’s body and mouth. Studies suggest that the reasons are likely to be a weakened immune system, poor nutrition, reduced salivary pH, and insufficient vitamin intake .

Saliva is a sticky, viscous oral fluid produced by three pairs of major and hundreds of minor salivary glands. It contains a wide range of compounds and physicochemical properties which are essential for maintaining oral and dental health [ 17 ]. Salivary buffering capacity is a critical factor in predicting oral pH and remineralization capacity [ 18 - 19 ]. Healthy saliva is either neutral or slightly alkaline which helps maintain one’s oral ecosystem in balance. It contains antimicrobial agents, including lysozyme, that protect the mouth against serious diseases [ 20 ]. The research conducted by Panagiotou *et al*. [ 21 ] (2021) showed that overweight or obese children had higher salivary cortisol levels, a reduced buffering capacity, and a decreased salivary flow.

Given the limited number of studies on the relationship between salivary biological markers and oral health and overweight and obesity in children, as well as the conflicting results in this regard, this study aimed to investigate the association between overweight and salivary bacterial and fungal populations in children.

## Materials and Method

### Study design and participants

This study was a cross-sectional comparative descriptive research conducted in 2021-2022 at two public schools in Qom City, Iran. The total sample size was calculated based on the study conducted by Araujo *et al*. [ 22 ] (2020) and using the formula for estimating sample size by Cochran i.e. considering a Type I error rate (alpha) of 5% and a Type II error rate (beta) of 10%. This resulted in a sample size of 90 participants (30 in each group). 

The inclusion criteria were defined as age group of 8-12 years; physically and mentally healthy children; parental consent for child participation; Iranian nationality; DMFT/dmft < 3. The exclusion criteria were taking medications, resistance, and lack of cooperation in collecting saliva and performing clinical examinations, and the consumption of antibiotics or any topical antibacterial solutions within the past 2 weeks.

After obtaining informed consent from parents and caregivers, 90 eligible children (45 males and 45 females) in three groups (15 normal weight, 15 overweight, and 15 obese children) were randomly selected from students who attended mentioned schools. The groups were matched based on age and gender.

### Data Collection and Sampling Method

Demographic information about the children and their parents as well as parental education levels were recorded in data collection forms. Information on the educational level of the children's parents was categorized as illiterate (1), primary school (2), high school diploma (3), bachelor's degree (4), master's degree (5), and PhD (6). Occupational groups were classified as unemployed (1), retired (2), self-employed (3), and government employees (4). The comparison of these groups was conducted by analyzing the frequency and rank of each group using non-parametric statistical tests.

The weight of children was measured with light clothes such as T-shirts and pants without shoes and belts using a digital scale (SBS 4414, sinbo, China) with an accuracy of 100 grams, and their height was measured using a wall meter with an accuracy of 0.5 cm. Then BMI was calculated by dividing weight in kilograms by the square of height in meters. After calculating the children’s BMIs based on the standard BMI chart designed by WHO [ 23 - 24 ], 90 qualified children were included in the study and divided equally into three groups of 30 people; children with normal weight, overweight children, and obese ones. According to WHO standards for children and adolescents aged 2 to 20 years, a BMI of 85% to 97% is considered as overweight, and a BMI above 97% is considered as obese.

### Saliva sample collection

After completing the questionnaire, the unstimulated saliva samples were collected from the participants in the morning before eating breakfast, brushing, or washing their mouths. The saliva sampling was conducted by holding the head down for two to three minutes and pouring 2ml of unstimulated saliva into a special sterile container (passive drooling method) [ 25 ]. The samples were kept in dry ice and were immediately transferred to the microbiology laboratory of Qom University of Medical Sciences for bacteriological and fungal evaluation. 

### Measuring salivary *Streptococcus mutans* level

**Figure 1:**
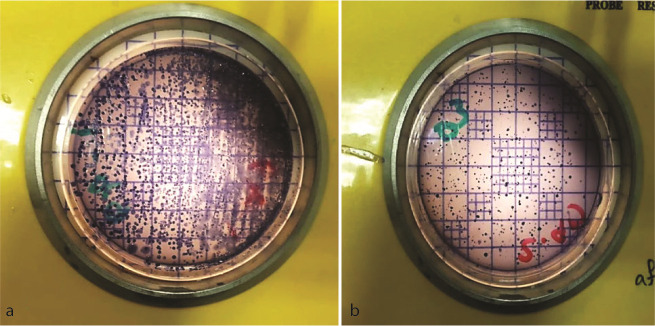
Colony count, scoring, and morphology of Lactobacillus and Streptococcus mutans. **a:** Streptococcus mutans: colonies were small, white and opaque. They exhibited a smooth, glistening surface with a dense growth pattern. **b:** Lactobacillus: colonies appeared gray, ranging in size from small to medium. The surface was smooth or slightly wrinkled with an irregular growth pattern

Then, 0.5ml of unstimulated saliva was combined with 5ml of phosphate-buffered saline (PBS) and was completely homogenized with a shaker. Then, 20 microliters of this mixture were added to the surface of the mitis salivarius agar medium with bacitracin and 10% sucrose. The plates were heated in an environment containing 5% CO2 at a temperature of 37℃ for 48 hours. Biochemical tests were carried out to isolate the *Streptococcus mutans* from other species such as mannitol, melibiose, sorbitol, and raffinose fermentation, and arginine dihydrolase test and Gram staining [ 26 - 28 ]. In the last step, the confirmed colonies of *Streptococcus mutans* were counted and scored (Figure 1).

### Measuring salivary *Lactobacillus* spp level

To isolate the *Lactobacillus* spp., a part of the sample was cultured in MRS broth liquid medium and kept for 48 hours at 37℃ under anaerobic conditions. Then the bacteria grown in the MRS broth medium were removed and cultured in the MRS agar medium. Because *Lactobacillus* spp. is an anaerobic microbe, it requires a special environment to grow. For anaerobic cultivation, the microbes were placed in an anaerobic jar (Merck, Darmstadt, Germany), a sealed container designed to prevent gas exchange with the external environment, ensuring suitable conditions for the growth of anaerobic bacteria. To create these conditions, a MERCK gas pack (Merck, Darmstadt, Germany), which is an oxygen absorbent chemical kit, was placed inside the jar. It was then soaked with 6 ml of normal saline and placed in an incubator with a temperature of 37℃. After keeping the samples in these environments for one day, a suspension was prepared from these microbes, similar to *Streptococcus mutans*. Differentiation of *Lactobacillus* spp colonies and its confirmation was conducted using morphological tests and Gram staining [ 29 ] (Figure 1).

Bacterial colonies were counted as A- zero colony: no growth; B- 1 colony: 1-103 bacterial isolates per milliliter of saliva; C- 2 colonies: 103-105 bacterial isolates per milliliter of saliva; and D- 3 colonies: more than 105 bacterial isolates per milliliter of saliva [ 29 ].

### Measuring salivary *Candida albicans* level

To isolate* Candida*, 0.1 ml of saliva was cultured in suburb chloramphenicol dextrose agar medium and heated at 37 ℃ for 24 to 48 hours, and after 48 hours, the presence of *Candida albicans* colonies was checked by microscopic examination test based on the colony color.

Colony counting was performed as A- zero colony = no growth; B-1 colony = 1-10 *Candida albicans* isolates per milliliter of saliva; C-2 colonies = 10-100 *Candida albicans* isolates per milliliter of saliva; and D-3 colonies = more than 100 *Candida albicans* isolates per milliliter of saliva [ 30 ].

### Measuring salivary pH

To check the pH of saliva, the saliva samples were examined immediately after sampling by a pH meter device that was previously calibrated by two substances with pH of 4 and 7. The electrode of the device was first washed with distilled water and then placed inside the sample. The saliva pH was shown up to two decimals [ 31 ].

### Bias

The risk of bias was limited by oral hygiene education 2 weeks before the commencement of the study as well as the assessment of children’s salivary microbiota, pH, and buffering capacity using reliable methods in an exclusive laboratory in Qom.

### Statistical Analysis

Data were analyzed using SPSS version 24.0. Chi-square, One-way ANOVA, Kruskal-Wallis, Mann-Whitney, T-test along with Post hoc tests, were employed as the appropriate tests for the present study. Non-parametric equivalents were used if necessary. Additionally, Spearman rank correlation coefficients were examined in the analyses.

### Ethical Considerations

The study was conducted with an Ethical Approval (IR.MUQ.REC.1401.135) from Qom University of Medical Sciences. Participation was voluntary, with no costs or penalties for withdrawal. Informed consent was obtained from both children (verbally) and their parents (written consent forms).

## Results

Ninety eligible children (45 boys and 45 girls), were selected for this study. Table 1 shows the demographic characteristics of participants.

**Table 1:** Demographic characteristics of children

Variable	Category	Number	Percentage (%)
Gender	Male	45	50.00
	Female	45	50.00
Obesity status (BMI)	Normal (<85th percentile)	30	33.33
	Overweight (85-97th percentile)	30	33.33
	obese (>97th percentile)	30	33.33
Age (in years)	8	16	17.80
	9	13	14.40
	10	23	25.60
	11	20	22.20
	12	18	20.00
Father's education	sub-diplomas	29	32.22
	diplomas	8	8.90
	higher diploma	53	58.88
Mother's education	sub-diplomas	35	38.90
	diplomas	25	27.77
	higher diploma	30	33.33

BMI: body mass index

The mean age was 10±1.4 years in the obese and normal-weight, and 10.16±1.39 years in the overweight groups. There was no significant difference based on gender (*p*= 1.00) or age (*p*= 0.98) between the groups. Also, there was no statistically significant relationship between the children's obesity status and parents' education level (Kruskal-Wallis, father’s *p*= 0.051, mother’s *p*= 0.862) (Table 2). 

**Table 2:** Relationship between parents' education and children's BMI

Parent's education	Status	Number	Mean Rank	*p* Value
father's education	Overweight	30	50.67	0.051
	Obese	30	40.58	
	Normal weight	30	36.25	
mother's education	Overweight	30	47.57	0.862
	Obese	30	44.35	
	Normal weight	30	44.58	

Kruskal-Wallis statistical analysis was conducted.

The significance level was set at 0.05.

The distribution of parental occupations was not significantly associated with the children's obesity status (Chi-square, father’s *p*= 0.224, mother’s *p*= 0.893) (Table 3). Additionally, there was no significant relationship between gender and children's BMIs. However, boys showed a slightly higher BMI compared to girls (Mann-Whitney U, *p*= 0.729) (Table 4).

**Table 3:** Relationship between children's obesity status and their parents' occupations

Parent's occupation	Status	Unemployed	Self-employed	Government-employed	Retired	Total	*p* Value
Father's occupation	Overweight	0	60%	26.66%	13.34%	100%	0.224
	Obese	0	56.66%	36.66%	6.68%	100%	
	Normal	0	80%	13.34%	6.66%	100%	
Mother's occupation	Overweight	0	10%	0	90%	100%	0.893
	Obese	0	6.66%	1.11%	90%	100%	
	Normal	0	1.11%	3.33%	86.66%	100%	

Chi-Square statistical analysis was conducted The significance level was set at 0.05

**Table 4:** Relationship Between Gender and Children's BMI

	Gender	Number	Mean	SD	*p* Value
BMI	Male	45	21.45	5.52	0.729
	Female	45	21.08	2.48	

T-test was conducted.

The significance level was set at 0.05.

(BMI: body mass index, SD: standard deviation)

According to Table 5, there was no significant correlation between the salivary colony count of *Lactobacillus* (*p*= 0.857), *Streptococcus mutans* bacteria (*p*= 0.777), and salivary pH (*p*= 0.639) in children (one-way ANOVA). Nevertheless, the salivary *Lactobacillus* bacteria colony count in obese children was higher than that of the other groups, and the salivary *Streptococcus mutans* bacteria colony count in overweight children was greater than that of the other groups.

**Table 5:** Relationship Between the Colony Count of Bacteria and Fungi and Salivary pH with Obesity Status in Children

	Status	Number	Mean	SD	*p* Value
*Lactobacillus*	Overweight	30	22.90	21.54	0.857
	Obese	30	25.53	19.77	
	Normal	30	22.86	22.61	
	Total	90	23.76	21.14	
*Streptococcus mutans*	Overweight	30	24.10	15.87	0.777
	Obese	30	21.33	17.01	
	Normal	30	21.40	18.58	
	Total	90	23.76	17.04	
*Candida albicans*	Overweight	30	4.33	6.59	0.046
	Obese	30	11.93	11.67	
	Normal	30	14.06	23.61	
	Total	90	10.11	16.05	
*Saliva pH*	Overweight	30	7.60	0.380	0.639
	Obese	30	7.51	0.554	
	Normal	30	7.51	0.353	
	Total	90	7.54	0.436	

One-way ANOVA analysis was conducted.

The significance level was set at 0.05. (SD: standard deviation)

However, there was a statistically significant relationship between salivary *Candida albicans* (CFU/ml) level and the children's obesity status. The mean salivary *Candida albicans* colony count (CFU/ml) was higher in normal-weight compared to all other groups, and it was lower in overweight children (one-way ANOVA, *p*= 0.046).

Table 6 presents the results of a Post hoc analysis for the relationship between *Candida albicans* and the obesity status of children, comparing normal-weight, overweight, and obese children. The results suggested that there was a statistically significant difference in terms of *Candida albicans* levels between the overweight and normal groups (Tukey’s HSD test, *p*= 0.048), while there was no such significant difference between the others.

**Table 6:** Post hoc analysis of the relationship between *Candida albicans* and the obesity status of children

					95% Confidence Interval	
Obesity Status	Obesity Status	Mean Difference	SD	*p* Value	Lower Bound	Upper Bound
Overweight	Obese	-7.60000	4.04784	0.151	-17.2520	2.0520
	Normal	-9.73333	4.04784	0.048	-19.3853	-0.0813
Obese	Overweight	7.60000	4.04784	0.151	-2.0520	17.2520
	Normal	-2.13333	4.04784	0.858	-11.7853	7.5187
Normal	Overweight	9.73333	4.04784	0.048	0.0813	19.3853
	Obese	2.13333	4.04784	0.858	-7.5187	11.7853

A Post Hoc Tukey’s HSD test was conducted with the Dependent Variable: *candida albicans*. The significance level was set at 0.05. (SD: standard deviation)

According to Table 7, a statistically significant correlation was observed between the levels of salivary *Candida albicans* and *Streptococcus mutans*. Individuals with a higher salivary *Streptococcus mutans* level tended to exhibit a significantly lower colony count of *Candida albicans* (*p*= 0.046, correlation coefficient= -0.211). There was a significant correlation between the levels of salivary *Lactobacillus* bacteria and *Candida albicans* (*p*= 0.002, Spearman correlation coefficient= 0.327).

**Table 7:** Correlation between salivary total bacterial colonies, fungal colonies, and salivary pH

		*Lactobacillus*	*Streptococcus mutans*	*Candida albicans*	Saliva pH
*Lactobacillus*	Correlation Coefficient	1.00	-0.033	0.327	-0.044
	*p Value*	0	0.756	0.002	0.679
	Total	90	90	90	90
*Streptococcus mutans*	Correlation Coefficient	-0.033	1.00	-0.211	-0.021
	*p Value*	0.756	0	0.046	0.846
	Total	90	90	90	90
*Candida albicans*	Correlation Coefficient	0.327	-0.211	1.00	0.023
	*p Value*	0.002	0.046	0	0.833
	Total	90	90	90	90
pH	Correlation Coefficient	-0.044	-0.021	0.023	1.00
	*p Value*	0.679	0.846	0.833	0
	Total	90	90	90	90

Nonparametric Spearman's Rank Correlation analysis performed. Correlation was significant at the 0.05 level

## Discussion

Childhood is a critical period for establishing healthy lifestyle habits that influence long-term weight management and overall health. These habits are closely linked to oral and dental health throughout life. Understanding the relationship between childhood obesity and risk factors for dental decay and periodontal diseases can provide valuable insights for preventing oral and systemic health issues in adulthood. This study found no significant associations between salivary pH, *Lactobacillus*, *Streptococcus mutans* levels, and BMI or obesity status in children. However, a negative correlation was seen between *Candida albicans* colony counts and childhood obesity. Additionally, a higher salivary Strep tococcus mutans level was linked to a lower *Candida albicans* count, indicating a potential inhibitory effect of *Streptococcus mutans* on *Candida albicans* growth.

However, obesity remains a pressing and persistent health concern, linked to an elevated risk of chronic illnesses [ 32 ]. Over the last decade, the global incidence of obesity has surged, influenced by multiple factors [ 5 ]. In our current study, no significant correlation was observed between parental education and occupation and childhood obesity. In line with our findings, Akbari *et al*. [ 33 ] (2006) did not find a significant link between parental education, maternal employment, and child gender with childhood obesity. In a related study conducted by Shahgholian *et al*. [ 34 ] (2004), no substantial association was observed between parental education and the prevalence of obesity. Conversely, a study conducted by Muthuri *et al*. [ 35 ] in 2016 revealed prominent relationships between parental education and childhood overweight across different countries (Colombia, Kenya, Brazil, and the United States). This research suggested that these conflicting findings may be attributed to racial, geographical, or sample size variations in these countries.

Oral microbiomes, including *Candida albicans*, *Streptococcus mutans*, and *Lactobacillus*, are known to contribute to the development of tooth decay through processes such as carbohydrate metabolism. Moreover, there is a direct link between obesity and an increased bacterial presence in both the oral and systemic environments. Furthermore, several studies have indicated a significant correlation between obesity and dental caries, with salivary buffering capacity and pH levels playing key roles [ 15 , 31 ].

In the present study, there was no statistically significant difference in salivary pH between obese, overweight, and normal-weight children. Notably, the overweight group showed the highest mean of salivary pH values. A similar finding was reported by de Campos *et al*. [ 36 ] (2014), who did not reveal a significant correlation between salivary pH and obesity status. Nonetheless, a study conducted by Bud *et al*. [ 37 ] (2021) did not identify any significant relationship in salivary pH, buffer capacity, or dental caries prevalence among underweight, normal-weight, and overweight children. However, it is worth noting that the underweight group showed significantly lower salivary pH levels.

Healthy saliva plays a crucial role in safeguarding the mouth against severe diseases due to its antimicrobial components, such as lysozyme [ 20 ]. As a result, salivary buffering capacity is a vital predictor of oral pH levels and the ability to remineralize teeth [ 18 , 19 ]. Recent research has indicated that elevated salivary cortisol levels in overweight and obese children can reduce buffering capacity and salivary flow, potentially leading to poorer oral health in overweight children when compared to those with a normal weight [ 21 ]. 

Nevertheless, there have been relatively few studies investigating the relationship between salivary pH and obesity, and conducting further research in this area seems to be essential [ 38 - 40 ]. 

Based on the findings of the current study, overweight children had lower salivary *Lactobacillus* levels (Table 5). However, no significant correlation was found between BMI and salivary *Lactobacillus* levels. Additionally, there was no significant correlation between BMI and the level of *Streptococcus mutans* in saliva. However, obese children generally had higher salivary *Streptococcus mutans* levels than other groups. Furthermore, a notable negative relationship was seen between childhood obesity and the number of salivary *Candida albicans* colonies. 

Regarding our findings, Mervish *et al*. [ 41 ] (2017) reported lower levels of salivary *Lactobacillus* in overweight children. In contrast, de Andrade *et al*. [ 42 ] (2020) did not find a significant relationship between BMI and salivary *Streptococcus mutans* in adolescents. However, Arvidsson *et al*. [ 43 ] (2015) showed a significant relation of salivary *Streptococcus mutans* level with BMI.

Consistent with our findings, Borgo *et al*. [ 44 ] (2017) reported a significantly lower prevalence of salivary* Candida* species in obese children. Moreover, according to the results of this study, Zakaria *et al*. [ 45 ] (2017) found that a lower BMI was associated with a higher likelihood of candidiasis among Japanese elderly individuals. Nonetheless, research on the alteration of salivary *Candida albicans* in children is limited, emphasizing the necessity of further investigations in this domain. They suggested that this relationship might be influenced by lifestyle changes, such as diet and smoking habits, which could impact the oral microbial balance [ 45 ].

In our current study, a significant correlation was observed between the levels of *Candida albicans*, *Streptococcus mutans*, and *Lactobacillus* in saliva. The results indicated that individuals with a higher abundance of *Streptococcus mutans* in their saliva tend to exhibit a reduced count of *Candida albicans*. Furthermore, our investigation revealed that as salivary *Lactobacillus* bacteria increases, the salivary *Candida albicans* count is elevated. These findings are consistent with those of the research conducted by Falsetta *et al*. [ 46 ] (2014), revealing a symbiotic relationship between *Streptococcus mutans* and *Candida albicans* in the oral microbiome, with glucosyltransferase acting as a mediator for their interaction. However, the study by Fujinami *et al*. [ 47 ] in 2021 reported a negative correlation between *Candida albicans* and, *Streptococcus mutans* and *Lactobacillus*.

In justifying this verity, researchers believe that these two key oral microorganisms, *Streptococcus mutans* and, *Lactobacillus*, can impact the growth of *Candida albicans* through multiple mechanisms. *Lactobacillus* is capable of generating an acidic environment by producing lactic acid, inhibiting *Candida albicans* growth and biofilm formation [ 48 - 49 ]. On the other hand, *Streptococcus mutans* plays a role in enhancing the immune system by promoting the production of antimicrobial peptides and cytokines. Additionally, both of these microorganisms can compete with *Candida albicans* for essential nutrients [ 50 ]. Altogether, these mechanisms are believed to work together in preventing *Candida albicans* overgrowth. As a consequence, the oral microbiome, a complex ecosystem of bacteria and fungi, interacts with each other in intricate ways. These interactions, influenced by dietary changes, metabolic alterations, and immune system dysfunction, can lead to imbalances between beneficial and harmful microorganisms . Moreover, the oral microbiome is interconnected with other microbial communities in the body, such as the gut microbiome. This bidirectional relationship between microbiomes can significantly correlate with obesity, particularly in children. Therefore, modulating the oral microbiome through interventions like probiotics could offer potential therapeutic avenues for obesity management and overall health improvement . 

One of the key strengths of our study lies in its novelty, as it marks the first investigation of its kind conducted on an Iranian population. Furthermore, in previous similar studies, there has been a limited focus on thoroughly examining the correlation between the three microbial species, especially salivary *Candida albicans*, and childhood obesity. Additionally, to eliminate potential confounders, this research opted for an equal gender distribution between the test and control groups.

### Research Limitations

The limitations of this study include the small sample size and the collection of samples from limited places. These constraints were due to the strict regulations and necessary approvals required by the education authorities for collecting samples. 

## Conclusion

This study revealed no significant differences in the levels of predominant oral bacterial species (*Streptococcus mutans* and *Lactobacillus*) or salivary pH among children in the three weight groups (normal weight, overweight, and obese). However, a significant negative correlation was observed between childhood obesity and *Candida albicans* colony counts, indicating that fungal populations varied with BMI status. Additionally, the results demonstrated a significant interplay between *Candida albicans*, *Streptococcus mutans*, and *Lactobacillus*, suggesting that obesity influences the oral microbial ecosystem. These findings highlight the need for further exploration of the role of salivary fungi in obesity-related oral and systemic health outcomes. 

## References

[1] Panuganti KK, Nguyen M, Kshirsagar RK, Doerr C Obesity (Nursing) [Updated 2023 Aug 8]. In: StatPearls [Internet]. Treasure Island (FL): StatPearls Publishing; 2023 Jan-. https://www.ncbi.nlm.nih.gov/books/NBK568702/.

[2] Mohajeri A, Berg G, Watts A, Cheever VJ, Hung M ( 2024). Obesity and Dental Caries in School Children. J Clin Med.

[3] Blüher M ( 2019). Obesity: global epidemiology and pathogenesis. Nat Rev Endocrinol.

[4] McPhee PG, Singh S, Morrison KM ( 2020). Childhood obesity and cardiovascular disease risk: working toward solutio-ns. Can J Cardiol.

[5] Lin TK, Teymourian Y, Tursini MS ( 2018). The effect of sugar and processed food imports on the prevalence of overwe-ight and obesity in 172 countries. Global Health.

[6] Akbari H, Mohammadi M ( 2022). The Prevalence of Obesity in Iranian Children: A Systematic Review and Meta-analysis. J Pediatr Rev.

[7] Heerman WJ, Sommer EC, Slaughter JC, Samuels LR, Martin NC, Barkin SL ( 2019). Predicting early emergence of childhood obesity in underserved preschoolers. J Pediatr.

[8] Cvanova M, Ruzicka F, Kukletova M, Lipovy B, Gachova D, Izakovicova Holla L, et al ( 2022). Candida species and selected behavioral factors co-associated with severe early childhood caries: Case-control study. Front Cell Infect Microbiol.

[9] Isticato R, Ricca E ( 2014). Spore Surface Display. Microbiol Spectr.

[10] Sufaru IG, Lazar L, Sincar DC, Martu MA, Pasarin L, Luca EO, et al ( 2022). Clinical effects of locally delivered lactobacillus reuteri as adjunctive therapy in patients with periodontitis: A split-mouth study. Appl Sci.

[11] Kim K, Han K, Yang S ( 2020). Association between overweight, obesity and incidence of advanced dental caries in South Korean adults: A 10-year nationwide population-based observational study. PLoS One.

[12] Ndanu TA, Aryeetey R, Sackeyfio J, Otoo G, Lartey A, Opintan JA, et al ( 2015). Streptococcus mutans and Lactoba-cillus species infection in obese and non-obese school children in Accra, Ghana. J Obes Overweig.

[13] Srivastava N, Ellepola K, Venkiteswaran N, Chai LYA, Ohshima T, Seneviratne CJ ( 2020). Lactobacillus Plantarum 108 Inhibits Streptococcus mutans and Candida albicans Mixed-Species Biofilm Formation. Antibiotics (Basel).

[14] Bud ES, Bica CI, Stoica OE, Vlasa A, Eșian D, Bucur SM, et al ( 2021). Observational study regarding the relationship between nutritional status, dental caries, mutans streptococci and lactobacillus bacterial colonies. Int J Environ Res Public Health.

[15] Xiang Z, Wakade RS, Ribeiro AA, Hu W, Bittinger K, Simon-Soro A, et al ( 2023). Human Tooth as a Fungal Niche: Candida albicans Traits in Dental Plaque Isolates. mBio.

[16] Du Q, Ren B, He J, Peng X, Guo Q, Zheng L, et al ( 2021). Candida albicans promotes tooth decay by inducing oral microbial dysbiosis. ISME J.

[17] Pedersen AML, Sørensen CE, Proctor GB, Carpenter GH, Ekström J ( 2018). Salivary secretion in health and disease. J Oral Rehabil.

[18] Dipalma G, Inchingolo F, Patano A, Guglielmo M, Palumbo I, Campanelli M, et al ( 2023). Dental erosion and the role of saliva: a systematic review. Eur Rev Med Pharmacol Sci.

[19] Martignon S, Roncalli AG, Alvarez E, Aránguiz V, Feldens CA, Buzalaf MAR ( 2021). Risk factors for dental caries in Latin American and Caribbean countries. Braz Oral Res.

[20] Farooq I, Bugshan A ( 2020). The role of salivary contents and modern technologies in the remineralization of dental enamel: a narrative review. F1000Res.

[21] Panagiotou E, Agouropoulos A, Vadiakas G, Pervanidou P, Chouliaras G, Kanaka-Gantenbein C ( 2021). Oral health of overweight and obese children and adolescents: a comparative study with a multivariate analysis of risk indicators. Eur Arch Paediatr Dent.

[22] Araujo DS, Klein MI, Scudine KGdO, de Sales Leite  L, Parisotto TM, Ferreira CM, et al ( 2020). Salivary microbiological and gingival health status evaluation of adolescents with overweight and obesity: a cluster analysis. Front Pediatr.

[23] Tyson N, Frank M ( 2018). Childhood and adolescent obesity definitions as related to BMI, evaluation and management options. Best Pract Res Clin Obstet Gynaecol.

[24] Thau L, Gandhi J, Sharma S Physiology, Cortisol. 2023 Aug 28. In: StatPearls [Internet]. Treasure Island (FL): StatPearls Publishing; 2024 Jan–.

[25] Navazesh M ( 1993). Methods for collecting saliva. Ann N Y Acad Sci.

[26] Yoo SY, Park SJ, Jeong DK, Kim KW, Lim SH, Lee SH, et al ( 2007). Isolation and characterization of the mutans streptococci from the dental plaques in Koreans. J Microbiol.

[27] Shklair I, Keene H ( 1974). A biochemical scheme for the separation of the five varieties of Streptococcus mutans. Arch Oral Biol.

[28] Mehdipour A, Ehsani A, Samadi N, Ehsani M, Sharifinejad N ( 2022). The antimicrobial and antibiofilm effects of three herbal extracts on Streptococcus mutans compared with Chlorhexidine 0.2% (in vitro study). J Life Med.

[29] Barani Karbasaki  F, Hossenzadeh H, Fazli Bazzaz  BS, Hoda V, Ghazvini K ( 2016). Evaluation of antimicrobial effects of aqueous and alcoholic extracts of saffron on oral path-ogenic microbes (Streptococcus mutans, Lactobacillus, Candida albicans). J Mashhad Dent School.

[30] Nadig SD, Ashwathappa DT, Manjunath M, Krishna S, Annaji AG, Shivaprakash PK ( 2017). A relationship between salivary flow rates and Candida counts in patients with xerostomia. J Oral Maxillofac.

[31] Bakhshi M, Kavei D, Asayesh H, Mehdipour A ( 2015). Evaluating the effect of pregnancy on streptococcus mutans, ph and buffering capacity of saliva. Stud Med Sci.

[32] Kor M, Pouramir M, Khafri S, Ebadollahi S, Gharekhani S ( 2021). Association between Dental Caries, Obesity and Salivary Alpha Amylase in Adolescent Girls of Babol City, Iran-2017. J Dent.

[33] Akbari N, Forozandeh N, Delaram M, Rahimi M (2006). Parent’s perception of obesity their 6-12 year old in obese child can parental education be effective?. Iran J Endocrinol Metab.

[34] Shahgholian NAF, Deris F ( 2004). 90th percentile of Body Mass Index (BMI) and some obesity risk factors among 7-12 years old school children, Chaharmahal & Bakhtiary, 2002. J Shahrekord Univ Med Sci.

[35] Muthuri SK, Onywera VO, Tremblay MS, Broyles ST, Chaput JP, Fogelholm M, et al ( 2016). Relationships between parental education and overweight with childhood overweight and physical activity in 9–11 year old children: Results from a 12-country study. PloS One.

[36] de Campos MM, Kobayashi FY, Barbosa TD, Costa SD, Lucas BD, Castelo PM ( 2014). Characteristics of salivary secretion in normal-weight, overweight and obese children: a preliminary study: salivary composition and excessive fat tissue. Odontol.

[37] Bud ES, Bica CI, Stoica OE, Vlasa A, Eșian D, Bucur SM, et al (2021). Observational study regarding the relationship between nutritional status, dental caries, mutans streptococci, and lactobacillus bacterial colonies. Int J Environ Res Public Health.

[38] Akbarnejad AA, Mahjoub S, Tamadoni A, Masrour-Roudsari J, Seyedmajidi S, Ghasempour M ( 2022). Salivary oxidative stress, total protein, iron and pH in children with β-thalassemia major and their correlation with dental caries. J Dent.

[39] Alqaderi H, Hegazi F, Al-Mulla F, Chiu CJ, Kantarci A, Al-Ozairi E, et al ( 2022). Salivary Biomarkers as Predictors of Obesity and Intermediate Hyperglycemia in Adolescents. Front Public Health.

[40] Hatipoglu O, Maras E, Hatipoglu FP, Saygin AG ( 2022). Saliv-ary flow rate, pH, and buffer capacity in the individuals with obesity and overweight; A meta-analysis. Niger J Clin Pract.

[41] Mervish NA, Hu J, Hagan LA, Arora M, Frau C, Choi J, et al ( 2019). Associations of the Oral Microbiota with Obesity and Menarche in Inner City Girls. J Child Obes.

[42] de Andrade PAM, Giovani PA, Araujo DS, de Souza AJ, Pedroni-Pereira A, Kantovitz KR, et al ( 2020). Shifts in the bacterial community of saliva give insights on the relationship between obesity and oral microbiota in adolescents. Arch Microbiol.

[43] Arvidsson L, Birkhed D, Hunsberger M, Lanfer A, Lissner L, Mehlig K, et al ( 2016). BMI, eating habits and sleep in relation to salivary counts of mutans streptococci in children–the IDEFICS Sweden study. Public Health Nutr.

[44] Borgo F, Verduci E, Riva A, Lassandro C, Riva E, Morace G, et al ( 2017). Relative abundance in bacterial and fungal gut microbes in obese children: a case control study. Child Obes.

[45] Zakaria M, Furuta M, Takeshita T, Shibata Y, Sundari R, Eshima N, et al ( 2017). Oral mycobiome in community‐dwelling elderly and its relation to oral and general health conditions. Oral Dis.

[46] Falsetta ML, Klein MI, Colonne PM, Scott-Anne K, Gregoire S, Pai CH, et al ( 2014). Symbiotic relationship between Streptococcus mutans and Candida albicans synergizes virulence of plaque biofilms in vivo. Infect Immun.

[47] Fujinami W, Nishikawa K, Ozawa S, Hasegawa Y, Takebe J ( 2021). Correlation between the relative abundance of oral bacteria and Candida albicans in denture and dental plaques. J Oral Biosci.

[48] Vazquez-Munoz R, Dongari-Bagtzoglou A ( 2021). Anticandidal activities by lactobacillus species: an update on mechanisms of action. Front Oral Health.

[49] Zeise KD, Woods RJ, Huffnagle GB ( 2021). Interplay between Candida albicans and lactic acid bacteria in the gastrointestinal tract: Impact on colonization resistance, microbial carriage, opportunistic infection, and host immunity. Clin Microbiol Rev.

[50] Černáková L, Rodrigues CF ( 2020). Microbial interactions and immunity response in oral Candida species. Future Microbiol.

[51] Hedayati Z, Khalafinejad F ( 2014). Relationship between body mass index, skeletal maturation and dental development in 6-to 15-year old orthodontic patients in a sample of Iranian population. J Dent.

[52] Sharifzadeh SS, Lotfali E, Lesan S, Farrokhnia T ( 2023). Antifungal Effect of Probiotic Lactobacillus casei on Drug-Resistant Oral Candida albicans Isolated from Patients with Hematological Malignancy: An In vitro Study. J Dent.

